# Language and Memory Reserve Mediate Protective Effects of Social Support on MCI or Dementia Risk in Older Women

**DOI:** 10.1093/geroni/igaa057.1531

**Published:** 2020-12-16

**Authors:** Andrew Petkus, Tara Gruenewald, Diana Youan, Xinhui Wang, Margaret Gatz, Mark Espeland, Jiu-Chiuan Chen

**Affiliations:** 1 USC Keck School of Medicine, Los Angeles, California, United States; 2 Chapman University, Orange, California, United States; 3 University of Southern California, Los Angeles, California, United States; 4 Wake Forest School of Medicine, Winston-Salem, North Carolina, United States; 5 Keck School of Medicine, Los Angeles, California, United States

## Abstract

More supportive social relationships are protective of cognitive decline in older adulthood. Although supportive social relationships are hypothesized to promote cognitive reserve (CR; the cognitive adaption to neuropathology), it is unknown whether CR mediates associations between social support and risk of developing mild cognitive impairment or dementia (MCI/dementia). Data from 815 women (aged 73-87 years) participating in the Women’s Health Initiative Memory Study-MRI cohort (WHIMS-MRI) and Women’s Health Initiative Study of Cognitive Aging (WHISCA) were analyzed to examine whether domain-specific estimates of CR mediate associations between social support and incident MCI/dementia risk. Women completed the Medical Outcomes Study Social Support Scale (MOS-SS) in 2004-2005, a structural MRI (sMRI) of the brain in 2005-06, and annual extensive neuropsychological examinations till 2018. CR (6-months after completing the MOS-SS) was estimated across different domains (e.g. verbal memory, figural memory, language, visuospatial, and attention) as the residual variance after regressing out effects of sMRI variables, sociodemographic factors, and measurement error. Structural equation models were constructed to examine whether CR mediate associations between social support and MCI/dementia risk while adjusting for covariates. Higher social support was associated with lower MCI/dementia risk (hazard ratio=0.85 per 1-SD;p=0.037), higher language reserve (standardized β=0.09;p=0.008) and verbal memory reserve (standardized β=0.08;p=0.025). Language and verbal memory reserve each significantly explained approximately 14% of the protective effect of social support. Findings illustrate the heterogeneous effect of social support on CR, highlighting the importance of language and verbal memory reserve as mediators of the association between social support and MCI/dementia risk.

